# Fibroblast Migration Is Regulated by Myristoylated Alanine-Rich C-Kinase Substrate (MARCKS) Protein

**DOI:** 10.1371/journal.pone.0066512

**Published:** 2013-06-19

**Authors:** Laura E. Ott, Eui Jae Sung, Adam T. Melvin, Mary K. Sheats, Jason M. Haugh, Kenneth B. Adler, Samuel L. Jones

**Affiliations:** 1 Department of Clinical Sciences, College of Veterinary Medicine, North Carolina State University, Raleigh, North Carolina, United States of America; 2 Center for Comparative Medicine and Translational Research, North Carolina State University, Raleigh, North Carolina, United States of America; 3 Department of Chemical and Biomolecular Engineering, North Carolina State University, Raleigh, North Carolina, United States of America; 4 Department of Molecular Biomedical Sciences, College of Veterinary Medicine, North Carolina State University, Raleigh, North Carolina, United States of America; Karolinska Institutet, Sweden

## Abstract

Myristoylated alanine-rich C-kinase substrate (MARCKS) is a ubiquitously expressed substrate of protein kinase C (PKC) that is involved in reorganization of the actin cytoskeleton. We hypothesized that MARCKS is involved in regulation of fibroblast migration and addressed this hypothesis by utilizing a unique reagent developed in this laboratory, the MANS peptide. The MANS peptide is a myristoylated cell permeable peptide corresponding to the first 24-amino acids of MARCKS that inhibits MARCKS function. Treatment of NIH-3T3 fibroblasts with the MANS peptide attenuated cell migration in scratch wounding assays, while a myristoylated, missense control peptide (RNS) had no effect. Neither MANS nor RNS peptide treatment altered NIH-3T3 cell proliferation within the parameters of the scratch assay. MANS peptide treatment also resulted in inhibited NIH-3T3 chemotaxis towards the chemoattractant platelet-derived growth factor-BB (PDGF-BB), with no effect observed with RNS treatment. Live cell imaging of PDGF-BB induced chemotaxis demonstrated that MANS peptide treatment resulted in weak chemotactic fidelity compared to RNS treated cells. MANS and RNS peptides did not affect PDGF-BB induced phosphorylation of MARCKS or phosphoinositide 3-kinase (PI3K) signaling, as measured by Akt phosphorylation. Further, no difference in cell migration was observed in NIH-3T3 fibroblasts that were transfected with MARCKS siRNAs with or without MANS peptide treatment. Genetic structure-function analysis revealed that MANS peptide-mediated attenuation of NIH-3T3 cell migration does not require the presence of the myristic acid moiety on the amino-terminus. Expression of either MANS or unmyristoylated MANS (UMANS) C-terminal EGFP fusion proteins resulted in similar levels of attenuated cell migration as observed with MANS peptide treatment. These data demonstrate that MARCKS regulates cell migration and suggests that MARCKS-mediated regulation of fibroblast migration involves the MARCKS amino-terminus. Further, this data demonstrates that MANS peptide treatment inhibits MARCKS function during fibroblast migration and that MANS mediated inhibition occurs independent of myristoylation.

## Introduction

Myristoylated alanine-rich C-kinase substrate (MARCKS) is a ubiquitously expressed protein kinase C (PKC) substrate that binds both actin and calmodulin (CaM) and regulates actin dynamics. MARCKS is cooperatively tethered to cell membranes by insertion of its myristoylated amino-terminus as well as electrostatic interactions between the basic effector domain of MARCKS and acidic phospholipids of the plasma membrane [1], [2]. Phosphorylation of MARCKS by PKC, or CaM binding, results in the release of MARCKS from the plasma membrane into the cytosol in a process called the “myristoyl-electrostatic switch” mechanism [3]. Dephosphorylation or release of CaM results in the ability of MARCKS to return to the plasma membrane. This membrane to cytosol shuttling, or bi-lateral translocation of MARCKS, has been associated with the reorganization of the actin cytoskeleton [4], [5], with various cellular processes regulated by MARCKS, including: endo- [6], exo- [7], and phagocytosis [8], [9], as well as cell migration [10], [11].

MARCKS is involved in regulation of motility in various cell types including fibroblasts [12], myoblasts [13], human embryonic kidney cells [14], human hepatic stellate cells [10], vascular smooth muscle cells [15], neutrophils [16], macrophages [17], mesenchymal stem cells [18] and various cancer cells [11], [19], [20]. One of the initial steps during cell migration is adherence of cells to the extracellular matrix, and a role for MARCKS in regulating such cell adhesion has been established. Expression of a mutated MARCKS in which the myristoyl-electrostatic switch mechanism is altered (thus inhibiting MARCKS bi-lateral translocation) resulted in abrogated cell adhesion and spreading [12], [13]. Glioblastoma multiforme cells that express a constitutively active variant of the epidermal growth factor receptor (EGFR) underwent decreased adhesion, spreading and invasion when transfected with a siRNA targeting MARCKS [20]. Additionally, MARCKS is localized to focal adhesions during α_5_ integrin myoblast attachment and spreading and silencing of MARCKS resulted in decreased myoblast spreading [21].

Recently, a unique reagent called MANS, a myristoylated cell permeant peptide corresponding to the first 24-amino acids of MARCKS, has been used to demonstrate a role for MARCKS, specifically its myristoylated amino-terminus, in regulating the migration of neutrophils [16], macrophages [17] and mesenchymal stem cells [18]. These results raised the question as to which aspect(s) of the MANS peptide, as well as the amino-terminus of MARCKS, could be involved in regulation of cell migration, with particular interest in amino-terminal myristoylation, given its role in membrane attachment [22], [23]. Fibroblasts, as opposed to neutrophils as previously described [16], were utilized in these experiments for two reasons. First, to determine if myristoylation of MANS is involved in regulating cell migration, a genetic structure-function analysis was performed. Fibroblasts are more suitable for these studies as they are a migratory cell type that are easily transfected, unlike terminally differentiated and difficult to transfect neutrophils. Second, fibroblasts were used in these experiments because they solely express MARCKS [25], [34] while neutrophils, a phagocytic leukocyte similar to macrophages, may express MARCKS-like protein (MLP; also called MARCKS-related protein (MRP) or MacMARCKS) in addition to MARCKS [24]–[26]. Both MARCKS and MLP have a myristoylated amino-terminus with approximately 50% homology [22] and are involved in the regulation of cell migration [12], [13], [27]. Thus, our previous work demonstrating that MANS peptide treatment inhibits neutrophil migration [16] does not rule out the possible involvement of MLP.

Herein, the MANS peptide was utilized to demonstrate that MARCKS is involved in the regulation of directed fibroblast migration, as measured by scratch wounding and PDGF-BB–induced chemotaxis assays. Further, siRNAs that target MARCKS were used to demonstrate that MARCKS expression is not essential to cell migration and that the MANS peptide specifically inhibits MARCKS function. Additional genetic structure-function analysis revealed that MANS peptide mediated inhibition of NIH-3T3 cell migration does not require the presence of the myristic acid moiety on the amino-terminus, as expression of MANS or unmyristoylated MANS (UMANS) C-terminal EGFP fusion proteins resulted in similar levels of attenuated migration. Taken together, the results of these studies support our previous findings that MARCKS regulates cell migration, although MARCKS expression is not essential to the process. These studies also demonstrate that the MANS peptide alters MARCKS function and that myristoylation of the MANS peptide is not required for MANS peptide-mediated inhibition of fibroblast migration.

## Results

### MANS Peptide Attenuates Migration of Fibroblasts on Fibronectin or Collagen Substrates

**Figure 1:**
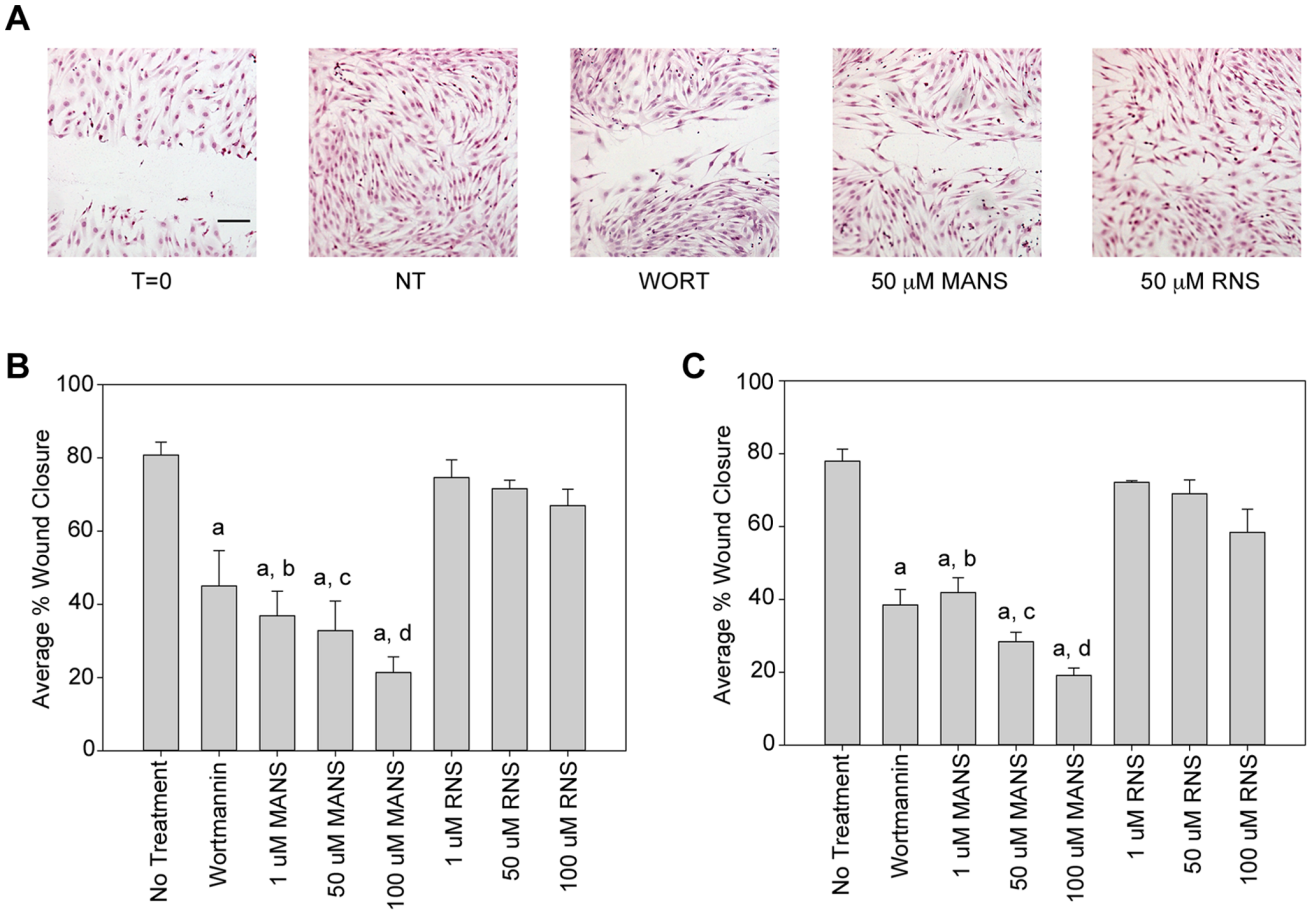
MANS peptide treatment attenuates migration of NIH-3T3 fibroblasts. NIH-3T3 fibroblasts were grown to confluency on fibronectin (A&B) or collagen (C) coated coverslips and scratch assays were performed with increasing concentrations (1, 50 or 100 µM) of MANS or RNS, VC (PBS) or 100 nM wortmannin. (A) Photos are representative of experiments on fibronectin-coated coverslips; bar is equivalent to 500 µm in length. The average percent wound closure is shown on fibronectin (B) or collagen substrates (C), with four individual experiments for both substrates performed. Statistical analysis (p<0.05) was performed where “a” denotes a significantly decreased ability to migrate back into the wound relative to no treatment and “b”, “c”, and “d” denote a statistically reduced ability to migrate back into the wound relative to 1 µM, 50 µM and 100 µM RNS, respectively.

MANS peptide treatment significantly decreased NIH-3T3 fibroblast migration on either fibronectin (Figures 1A & B) or collagen (Figure 1C) substrates in a concentration-dependent manner. Treatment of the cells with the myristoylated missense scrambled control (RNS) peptide did not alter the ability of fibroblasts to migrate on either fibronectin or collagen (Figure 1). As a positive control for inhibition of migration, fibroblasts were incubated with the phosphoinositide 3-kinase (PI3K) inhibitor wortmannin, which has been shown previously to inhibit NIH-3T3 fibroblast migration [28]. As expected, wortmannin treatment resulted in significantly decreased fibroblast migration compared to non-treated or RNS treated cells, while wortmannin and MANS treated cells had a similar level of inhibition (Figure 1). Taken together, these results are the first to demonstrate that the MANS peptide inhibits fibroblast migration and suggest that the amino-terminus of MARCKS may be involved in regulating this process. Based on these results, additional studies utilized a concentration of 50 µM MANS or RNS.

**Figure 2:**
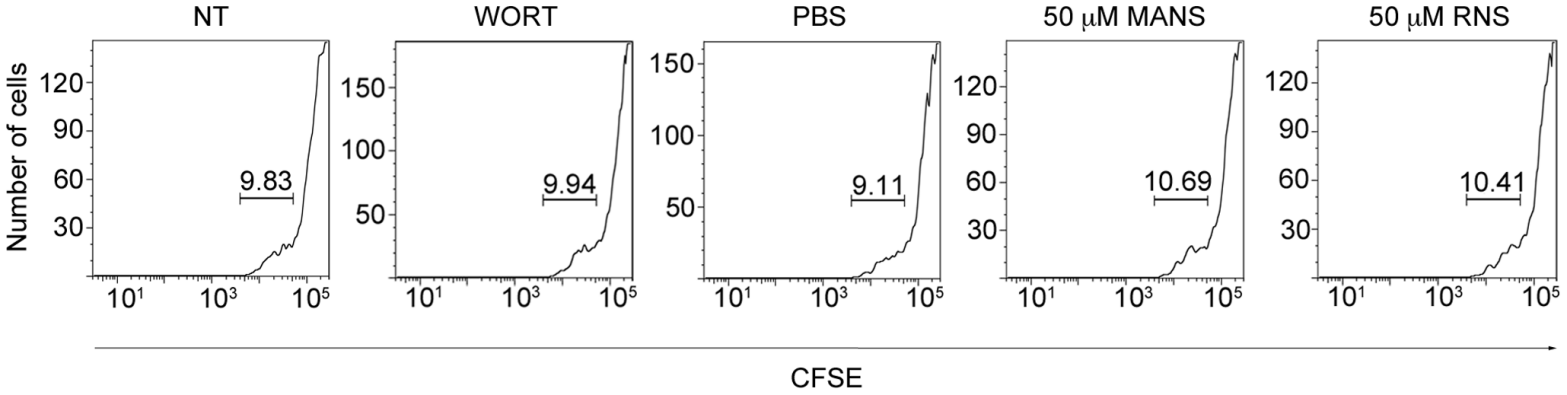
Fibroblast proliferation is not altered by MANS peptide treatment. CFSE labeled NIH-3T3 fibroblasts (5×10^5^ cells) were plated on fibronectin coated plates and allowed to adhere. Cells were incubated in DMEM with 2% FBS plus antibiotics in the presence of wortmannin (WORT; 100 nM), PBS (VC), 50 µM MANS or 50 µM RNS for 18 hours and cell proliferation was evaluated by flow cytometry. Data is representative of three independent experiments. NT denotes CFSE-labeled cells that were not treated.

To confirm that the results observed in our scratch-wounding assay were not due to altered cell proliferation, we performed carboxyfluorescein succinimidyl ester (CFSE) proliferation assays. Briefly, we incubated CFSE-labeled fibroblasts on fibronectin coated plates for 18 hours (duration of scratch assay) in the presence of 50 µM MANS, 50 µM RNS, vehicle control (VC; PBS) or wortmannin. As shown in figure 2, no difference was observed in NIH-3T3 proliferation in MANS or RNS treated cells compared to non-treated, wortmannin or VC treated cells. These results confirm that MANS peptide treatment does not alter cell proliferation within the context of our scratch assay and demonstrates that the results observed in Figure 1 are due to altered cell migration and not proliferation.

### MANS Inhibits PDGF-BB Stimulated Fibroblast Chemotaxis

**Figure 3:**
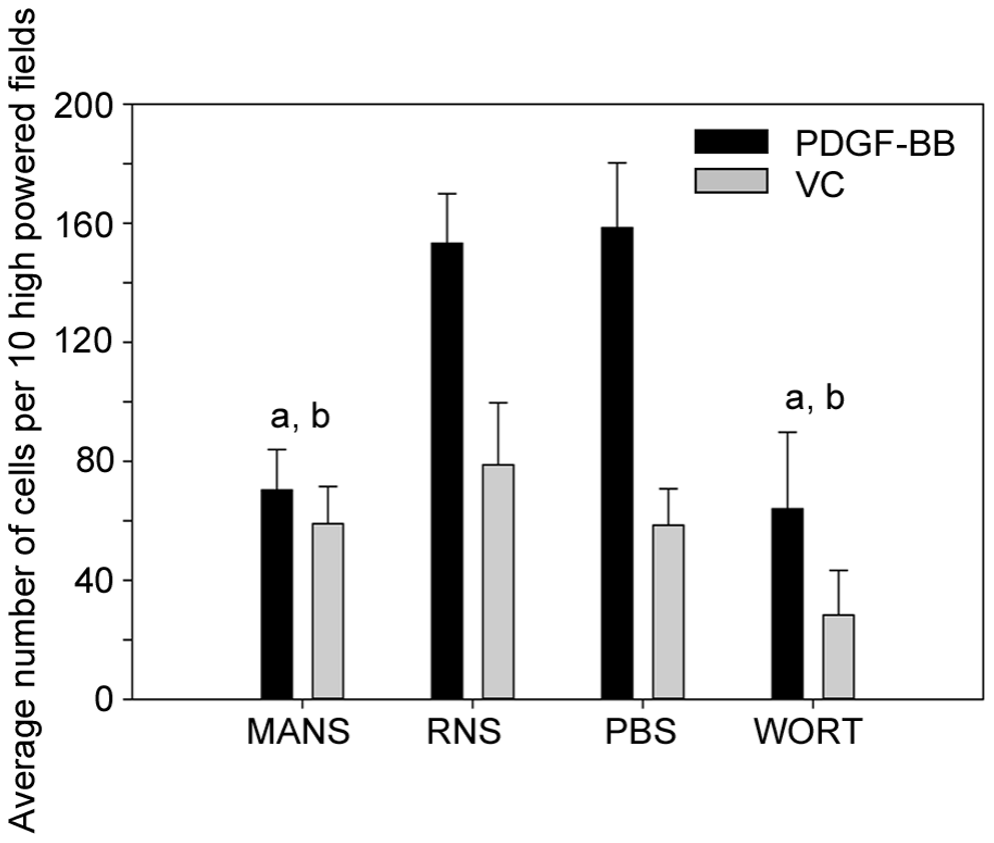
PDGF-BB mediated NIH-3T3 fibroblast chemotaxis is inhibited by MANS pretreatment. NIH-3T3 fibroblasts were pretreated with 50 µM MANS, 50 µM RNS, PBS (VC) or 100 nM wortmannin (WORT) for 30 minutes prior to adding the cells to fibronectin coated transwells with 1 nM PDGF-BB or VC (sterile water) in the bottom chamber. Transwell chambers were incubated for 4 hours and the transwell inserts were fixed, stained and mounted on microscope slides and the number of cells on the bottom side of the filter was counted in 10 high-powered fields. Data shown represents the average number of cells in 10 high-powered fields from four individual experiments with “a” and “b” denoting a significant decrease in percent (%) wound closure relative to RNS and VC treatment, respectively (p<0.05).

Fibroblasts migrate by both cell-contact cues, as demonstrated in the wound healing process, as well as by directional chemotaxis towards chemoattractants such as PDGF-BB [29]–[31]. In the past, PI3K has been thought to be the main mediator of cell migration, and several reports have established that PI3K is involved in regulating PDGF-BB mediated fibroblast migration [32]–[34]. However, PDGF-BB stimulation of Swiss 3T3 fibroblasts and human hepatic stellate cells also results in phosphorylation and membrane to cytosol translocation of MARCKS [10], [35]–[37], suggesting a role for MARCKS in PDGF-BB induced motility. To determine if MANS peptide treatment inhibits PDGF-BB mediated fibroblast migration, a Boyden chamber approach was utilized with fibronectin-coated transwells and PDGF-BB or vehicle control (VC; sterile water) placed in the bottom chamber. A concentration of 1 nM PDGF-BB was used for these studies as we have previously demonstrated this concentration to be optimal for inducing directional chemotaxis in NIH-3T3 fibroblasts [32]. As shown in Figure 3, pretreatment of cells with the MANS peptide attenuated fibroblast migration towards PDGF-BB compared to RNS peptide or PBS (VC) treatment. Similar to the scratch wounding assays, MANS peptide inhibition of migration was comparable to treatment with wortmannin (Figure 3). Interestingly, unstimulated migration in wells containing VC (sterile water) was not affected by MANS treatment, suggesting that MARCKS protein specifically regulates directed migration of fibroblasts.

### MANS Peptide Reduces Fibroblast Chemotaxis as Monitored by Live-cell Imaging

**Figure 4:**
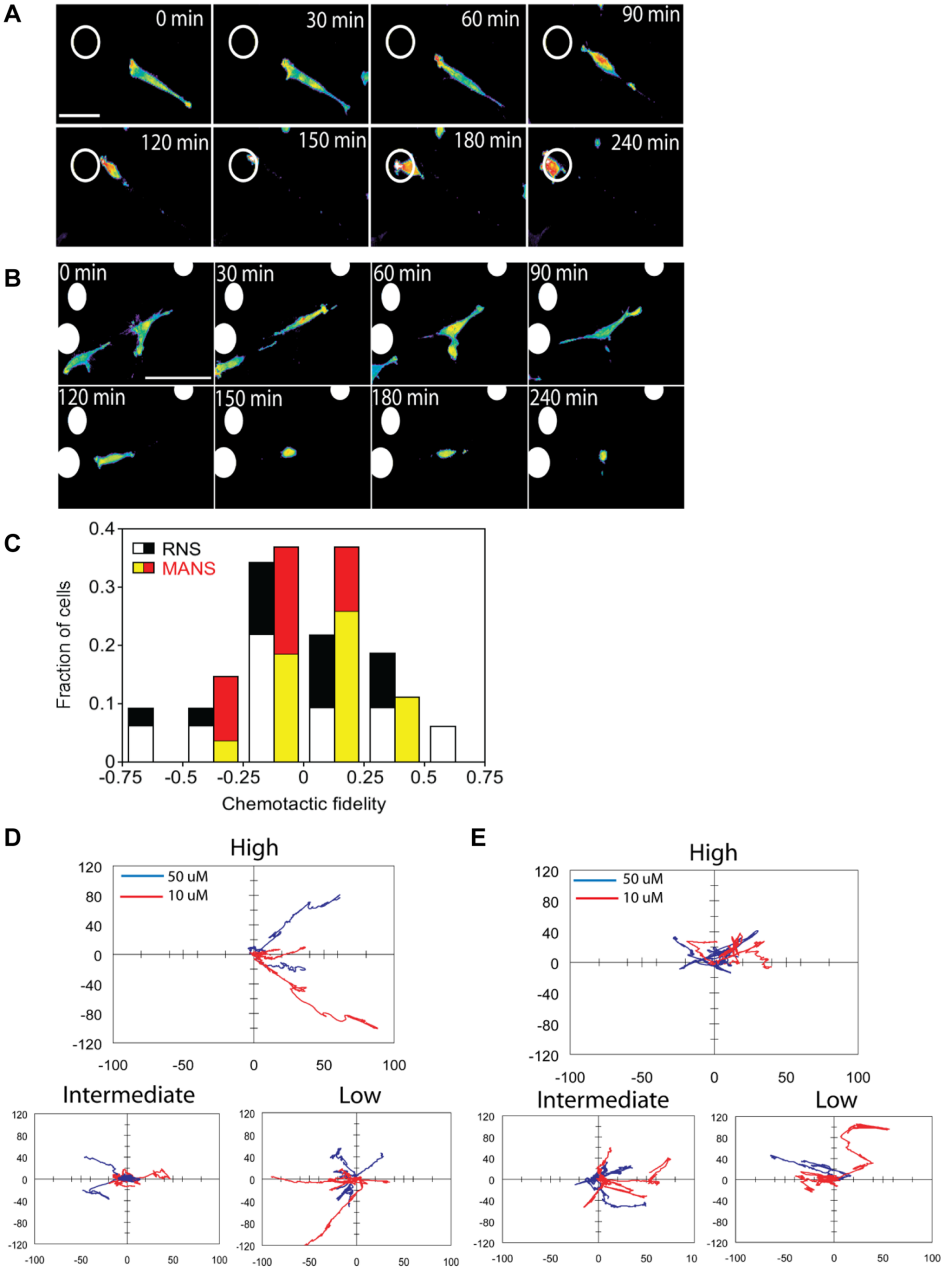
MANS inhibition of chemotaxis analyzed by live-cell TIRF microscopy. (A) Time-course montage of a GFP-AktPH-expressing NIH 3T3 mouse fibroblast migrating chemotactically in response to a PDGF-BB gradient emanating from an alginate microsphere (open circle). The cell was treated with RNS peptide and monitored by TIRF microscopy, with localization of PI3K signaling displayed using a pseudo-color intensity scale; scale bar = 100 µm. (B) Montage as in part A, except that incubation was in the presence of MANS peptide, which inhibited productive cell movement. Multiple microspheres in this field are indicated by filled circles; scale bar = 100 µm. (C) Chemotactic fidelity was quantified as the fraction of time intervals during which cell movement was aligned with the PDGF gradient (angle within 60°) minus the fraction of time intervals during which movement was misaligned (angle between 120–180°). The histogram compares cells incubated with RNS (*n* = 32) versus MANS (*n* = 27) at peptide concentrations of 10 µM (gray, yellow) and 50 µM (black, red). (D&E) For each of the 4 treatment conditions compared in part C, chemotactic fidelity values were sorted into high, medium, and low subpopulations for RNS (D) and MANS (E). Cell centroid translocation paths are plotted with the initial centroid positions located at the origin and the initial PDGF-BB gradient vector aligned along the positive *x*-axis.

To further examine how MANS peptide treatment inhibits MARCKS function and attenuates fibroblast chemotaxis, we used live-cell imaging by total internal reflection fluorescence (TIRF) microscopy. NIH-3T3 fibroblasts expressing GFP-AktPH, a fluorescent biosensor for PI3K signaling, were used to simultaneously monitor cell movement and polarity of intracellular signaling in response to PDGF-BB gradients [32]. Whereas cells treated with RNS control peptide exhibited normal morphology and migration response, cells treated with MANS peptide tended to exhibit smaller contact areas and reduced motility (Figures 4A & B). Chemotactic fidelity was quantified as the fraction of time the cell was moving towards the PDGF gradient (angle within 60°) less the fraction of time moving away from the gradient (angle between 120° and 180°); by this measure, the population of cells treated with MANS was underrepresented in cells exhibiting high fidelity (Figure 4C). Interestingly, the MANS-treated population was also underrepresented in cells moving predominantly away from the gradient, suggesting a general defect in cell migration persistence. Further analysis showed that, among the cells exhibiting the highest chemotactic fidelity in each population, the MANS-treated cells exhibited markedly less displacement from their original starting positions (Figures 4D & E). Interestingly, there were no differences observed in PI3K signaling as measured by the GFP-AktPH biosensor in either MANS or RNS treated cells. Taken together, these results confirm our Boyden chamber experiments (Figure 3) and demonstrate that MARCKS is involved in the directional migration of NIH-3T3 fibroblasts.

### MANS Peptide does not Alter PDGF-BB-induced MARCKS or AKT Phosphorylation

**Figure 5:**
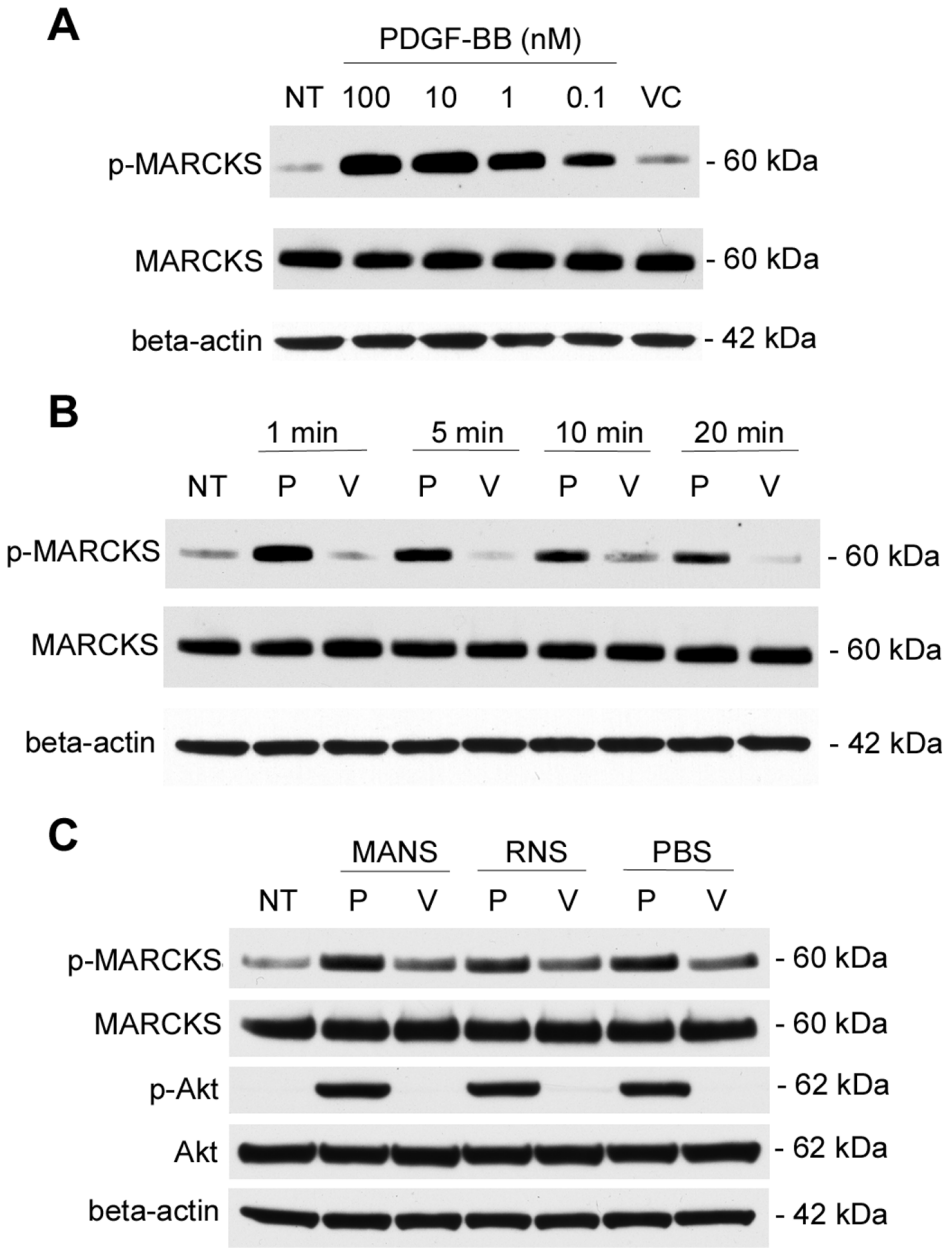
MANS pretreatment does not alter PDGF-BB mediated MARCKS phosphorylation or PI3K signaling. (A and B) Adherent fibroblasts were stimulated with the indicated concentrations of PDGF-BB for the indicated times and Western Blot analysis for phosphorylated MARCKS (p-MARCKS), total MARCKS (MARCKS) and beta-actin was performed. (A) Representative dose-response (1 min stimulation) experiment. (B) Representative kinetics analysis (10 nM PDGF-BB) experiment. (C) Adherent fibroblasts were pretreated with 50 µM MANS, 50 µM RNS or PBS (VC for MANS or RNS peptides) for 30 minutes prior to stimulation with 10 nM PDGF-BB or VC (sterile water) for 1 minute. Western blot analysis was performed to determine the expression of phosphorylated MARCKS (p-MARCKS), total MARCKS (MARCKS), phosphorylated AKT1 (p-AKT), total AKT (AKT) and beta-actin. NT denotes no treatment and VC denotes vehicle control (sterile water); data is representative of three separate experiments.

PDGF-BB stimulation results in phosphorylation of MARCKS in Swiss 3T3 fiboroblasts [34]–[36] as well as in human hepatic stellate cells [10]. Given that phosphorylation of MARCKS and subsequent membrane to cytosolic translocation is associated with MARCKS function, we asked if MANS peptide inhibition of PDGF-BB mediated chemotaxis was due to altered MARCKS phosphorylation. To address this question, we performed initial experiments to determine the conditions for optimal PDGF-BB induced MARCKS phosphorylation in fibronectin adherent NIH-3T3 fibroblasts. First, we performed a dose response assay by stimulating cells with 100, 10, 1, or 0.1 nM PDGF-BB for 1 minute and found 10 nM to be the optimal concentration of PDGF-BB to stimulate MARCKS phosphorylation (Figure 5A). Next, we performed a kinetics analysis by stimulating cells with 10 nM PDGF-BB for 1, 5, 10, or 20 minutes and found a 1 minute stimulation with 10 nM PDGF-BB resulted in optimal MARCKS phosphorylation (Figure 5B). To address if MANS peptide treatment alters PDGF-BB induced MARCKS phosphorylation, fibronectin adherent NIH-3T3 fibroblasts were pretreated with MANS, RNS or PBS (VC) for 30 min and then stimulated with 10 nM PDGF-BB for 1 min. As shown in Figure 5C, MANS pretreatment did not alter MARCKS phosphorylation in either VC or PDGF-BB stimulated NIH-3T3 cells, as no difference in MARCKS phosphorylation was observed in MANS, RNS or PBS treated cells. This demonstrates that MANS peptide inhibition of PDGF-BB induced fibroblast motility is not due to alterations in MARCKS phosphorylation.

To determine whether MANS peptide treatment interferes with PDGF-BB mediated PI3K signaling, we evaluated phosphorylation of Akt in cells stimulated with PDGF-BB that were treated with MANS or RNS. As shown in Figure 5C, no differences in Akt phosphorylation were observed in MANS, RNS or PBS treated cells upon PDGF-BB or VC stimulation. These results support the findings of the GFP-AktPH biosensor live-cell imaging experiment (Figure 4) and demonstrate that MANS peptide treatment does not interfere with PDGF-BB induced PI3K signaling.

### Expression of MARCKS is not Required for NIH-3T3 Fibroblast Migration

**Figure 6:**
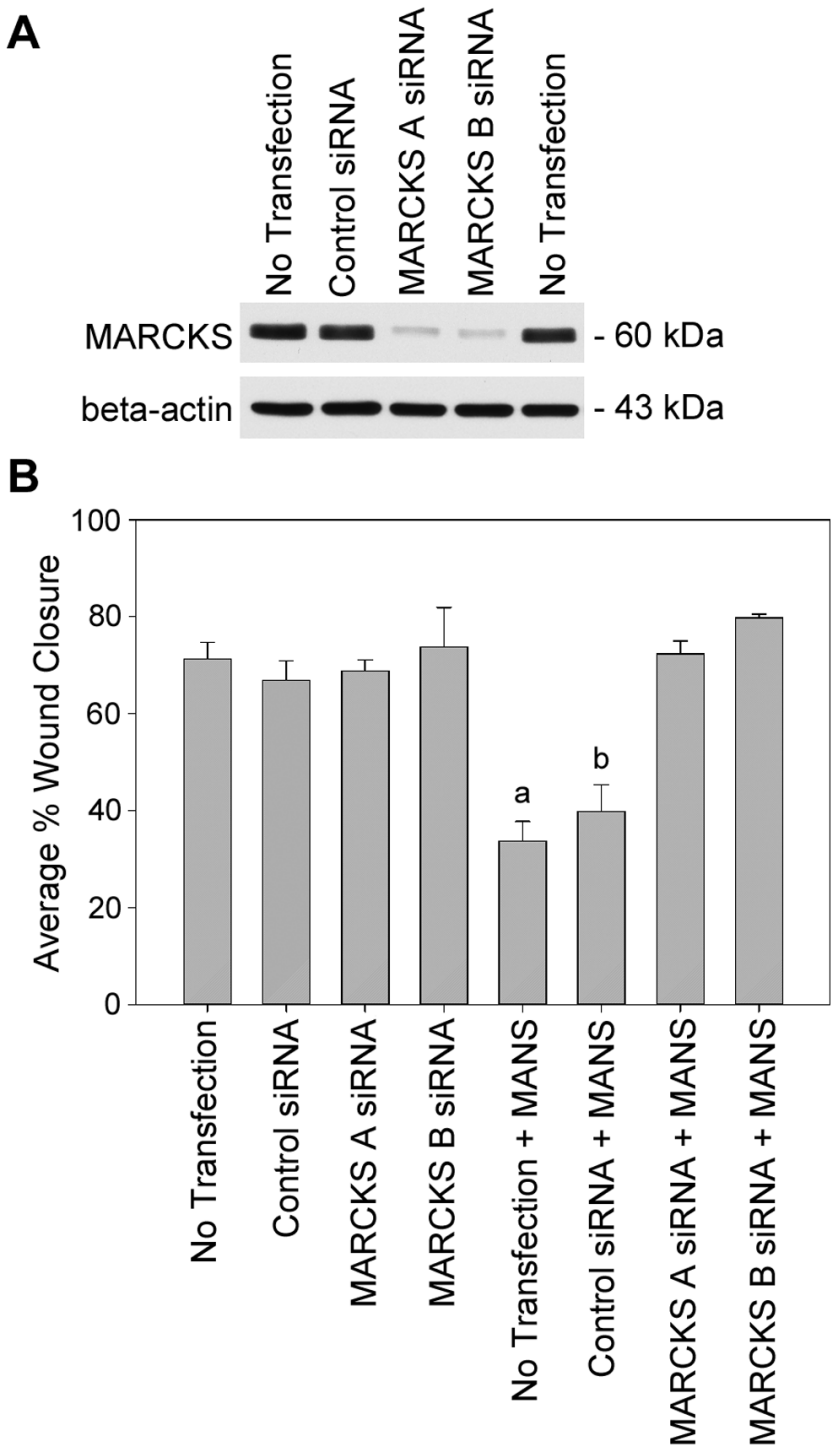
MARCKS protein expression is not essential to NIH-3T3 fibroblast migration. (A) NIH-3T3 cells were transfected with control, MARCKS A or MARCKS B siRNAs and total MARCKS and beta-actin expression was determined by Western blot 48 hours post transfection. (B) Scratch-wounding assays on non-transfected, control siRNA, MARCKS A siRNA or MARCKS B siRNA transfected cells were performed with or without 50 µM MANS treatment. Statistical analysis (p<0.05) was performed where “a” denotes a significantly decreased ability to migrate back into the wound relative to non-transfected cells and “b” denotes significantly decreased ability to migrate back into the wound relative to control siRNA transfected cells. Data is representative of three independent experiments.

To determine whether MARCKS expression is essential to NIH-3T3 fibroblast migration, we transfected cells with two separate siRNAs targeting MARCKS and performed scratch-wounding assays on a fibronectin substrate. MARCKS knockdown was observed 48 hours after transfection with either MARCKS A siRNA or MARCKS B siRNA relative to control siRNA transfected or non-transfected cells (Figure 6A). Equal expression of beta-actin was observed in non-treated cells as well as control and MARCKS siRNA treated cells (Figure 6A). As shown in Figure 6B, no difference in cell migration was observed in non-transfected cells or cells transfected with control siRNA, MARCKS A siRNA or MARCKS B siRNA, suggesting that expression of MARCKS is not essential for migration of NIH-3T3 fibroblasts.

We next evaluated if MANS peptide treatment altered NIH-3T3 fibroblast migration in cells with attenuated MARCKS expression. We stimulated non-transfected or control siRNA, MARCKS A siRNA or MARCKS B siRNA transfected cells with MANS peptide to address this question. While MANS peptide treatment attenuated migration in non-transfected and control siRNA transfected cells, no difference in cell migration was observed in MARCKS A siRNA or MARCKS B siRNA transfected cells that were treated with or without MANS peptide (Figure 6B). Interestingly, reduced cell spreading was observed in control siRNA transfected cells treated with MANS peptide, whereas normal spreading was observed in MARCKS siRNA transfected cells with or without MANS peptide treatment (data not shown). Further, no difference in cell proliferation was observed within the parameters of the scratch assay in control siRNA, MARCKS A siRNA or MARCKS B siRNA transfected cells with or without MANS peptide treatment (data not shown). Taken together, this data demonstrates that MARCKS expression is not essential for NIH-3T3 fibroblast migration and further demonstrates that the MANS peptide specifically inhibits the function of MARCKS.

### Myristoylation of MANS is not Required for Inhibition of Fibroblast Migration

The MANS peptide is identical to the amino-terminus of MARCKS, including the presence of a myristic acid moiety. Myristoylation can enhance the ability of peptides to translocate across cell membranes (likely promoting the efficacy of the MANS peptide approach to alter MARCKS function in living cells) and contribute to peptide membrane attachment, which may be involved in the mechanism by which MANS affects MARCKS function. We could not determine the role for the myristic acid moiety in the latter mechanism without altering for former, so the role of myristoylation in MANS ability to alter MARCKS function and fibroblast function was addressed using a genetic structure-function approach. We generated pEGFP-N1 expression plasmids encoding C-terminal EGFP fusion proteins of either the MANS sequence or a G2A point mutant that eliminates the myristoylation signal, resulting in unmyristoylated MANS (UMANS). The parent pEGFP-N1 vector served as a control plasmid for these experiments rather than RNS::EGFP. Since RNS is a myristoylated peptide with the same amino acid composition as MANS but in a random scrambled sequence, cloning the RNS sequence into pEGFP-N1 using traditional methods would be challenging. Additionally, myristoyl-CoA:protein N-myristoyltransferase myristoylates proteins at the amino acid consensus sequence is NH2-GXXXS. While both MANS and RNS peptides are commercially synthesized with an amino-terminal myristic acid, it is likely that only MANS would be myristoylated upon epigenetic expression as it has the sequence NH2-GAQFS. RNS, with a sequence of NH2-GTAPA, would likely not be myristoylated upon epigenetic expression as it lacks a serine in the in the fifth position of the myristoylation consensus sequence [38], [39]. Thus, we chose to use the parent pEGFP-N1 vector for the control of these experiments as there is a chance that RNS::EGFP would likely function differently than the RNS peptide.

**Figure 7:**
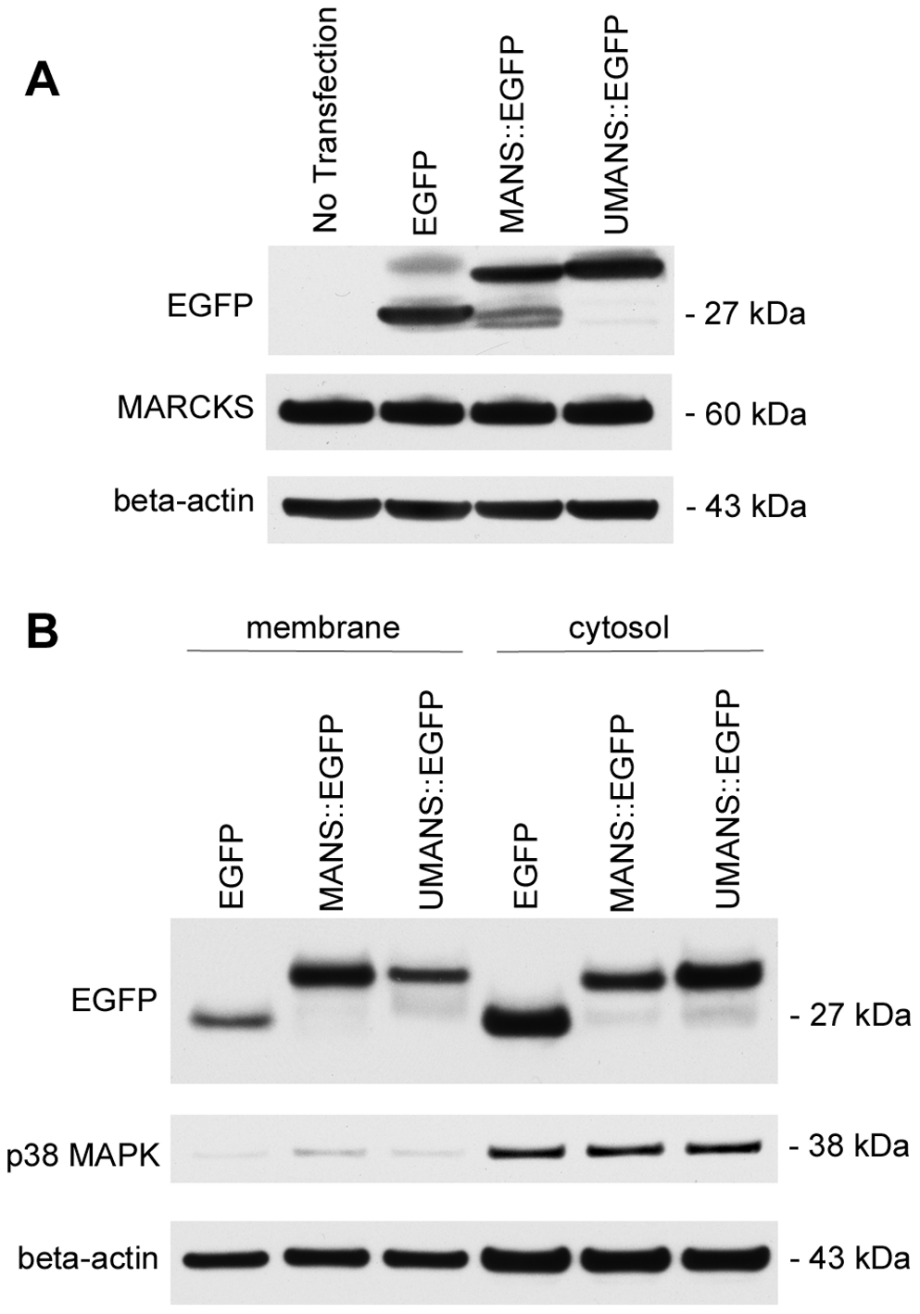
Expression and cellular localization of MANS::EGFP and UMANS::EGFP fusion proteins. (A) Transfection of EGFP, MANS::EGFP and UMANS::EGFP results in similar expression of EGFP in NIH-3T3 fibroblasts as determined by Western blot 24 hours post transfection, with no expression of EGFP in non-transfected cells. Total MARCKS and beta-actin (loading control) expression in transfected and non-transfected NIH-3T3 fibroblasts was also determined 24 hours post transfection. (B) Analysis of membrane and cytosolic fractions for the expression of EGFP, MANS::EGFP and UMANS::EGFP as determined by Western blot analysis for EGFP expression. Cytosolic fraction purity was determined by p38 MAPK expression and beta-actin served as a loading control. Figures are representative of three independent experiments.

Western blot analysis using an anti-EGFP antibody demonstrated equal expression of EGFP, MANS::EGFP and UMANS::EGFP proteins 24-hours post transfection (Figure 7A). Western blot analysis also revealed equal expression of MARCKS in non-transfected, EGFP, MANS::EGFP and UMANS::EGFP transfected cells (Figure 7A). Subcellular fractionation revealed that MANS::EGFP appears to be predominantly targeted to the membrane fraction (with some cytosolic localization) while EGFP and UMANS::EGFP are predominantly localized to the cytosol (Figure 7B). Interestingly, in spite of the fact that UMANS::EGFP does not contain a myristoylation signal, the fusion protein did appear to associate with the membrane fraction, albeit less so than MANS::EGFP. As a control for fraction purity and equal protein loading, the expression of p38 MAPK (cytosolic fraction only) and beta-actin were also determined, respectively.

**Figure 8:**
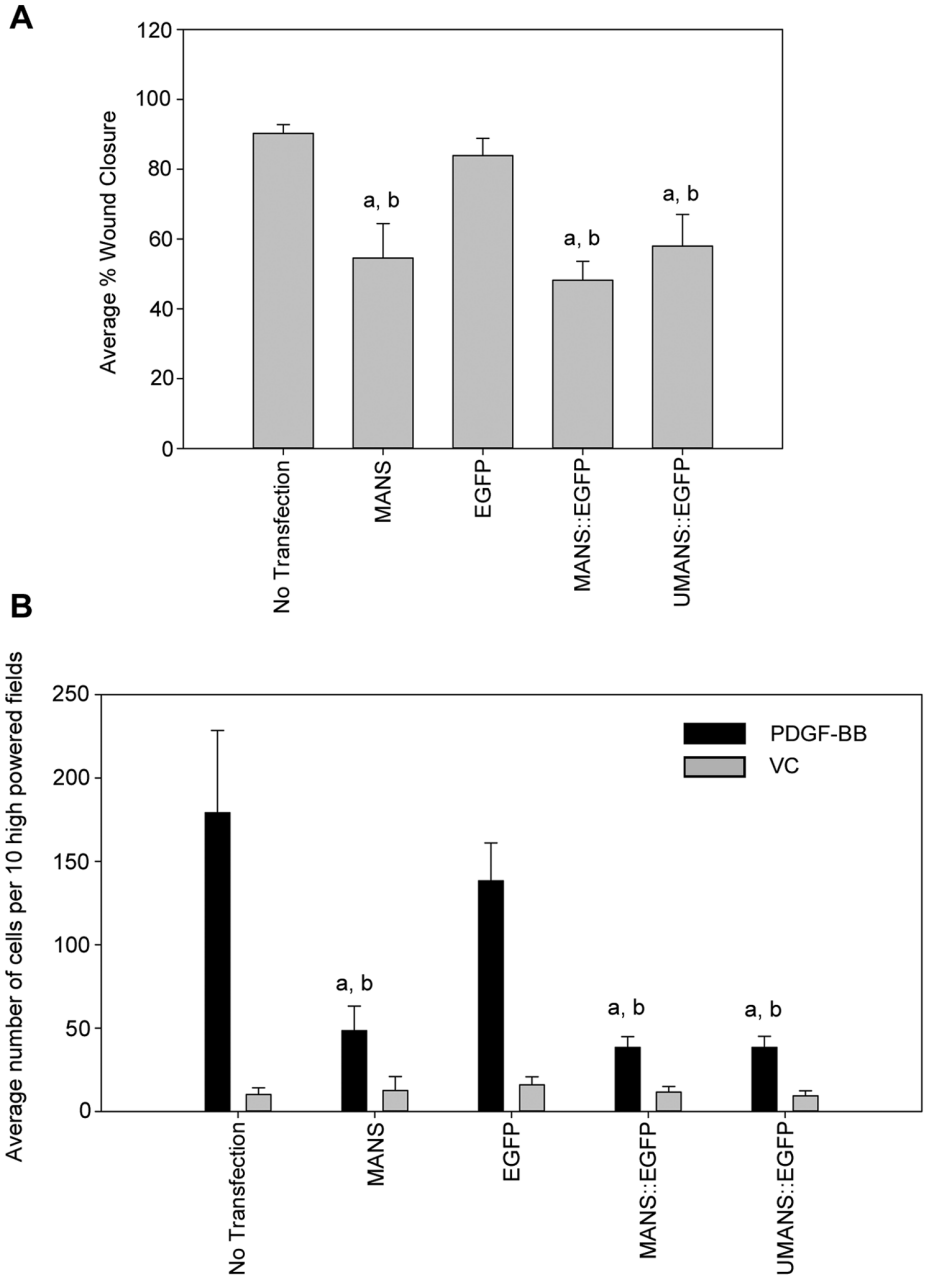
Myristoylation of MANS is not required for attenuation of fibroblast migration. (A) Transfected (EGFP, MANS::EGFP or UMANS::EGFP) or non-transfected NIH-3T3 fibroblasts were plated on fibronectin coated coverslips and scratch assays were performed as described. Non-transfected cells were treated with the MANS peptide (50 µM) as a positive control for inhibition of cell migration. (B) PDGF-BB chemotaxis assays were performed with NIH-3T3 fibroblasts that were non-transfected or transfected with EGFP, MANS::EGFP or UMANS::EGFP as described. Non-transfected cells were treated with the MANS peptide (50 µM) as a positive control for inhibition of cell migration. For both experiments, “a” and “b” designate statistically significant differences relative to non-transfected or EGFP transfected cells, respectively (p<0.05), and data is representative of five independent experiments.

We next determined whether expression of MANS::EGFP affected fibroblast migration in a similar manner to MANS peptide treatment. Evaluation of NIH-3T3 cell migration using the scratch-wounding assay demonstrated that expression of MANS::EGFP significantly decreased migration compared to non-transfected cells and cells expressing EGFP alone. Further, expression of MANS::EGFP attenuated migration to a level approximately equal to that of 50 µM MANS peptide treatment (Figure 8A). Interestingly, expression of UMANS::EGFP also significantly inhibited NIH-3T3 fibroblast migration, similar to MANS peptide treated cells or cells expressing MANS::EGFP (Figure 8A). Further, expression of either MANS::EGFP or UMANS::EGFP in NIH-3T3 cells significantly inhibited migration in response to PDGF-BB in Boyden chamber chemotaxis assays compared to untreated cells and cells expressing EGFP alone (Figure 8B). As in the scratch assay, expression of MANS::EGFP and UMANS::EGFP fusion proteins inhibited PDGF-BB mediated fibroblast migration comparably to 50 µM MANS peptide treatment. There was no difference in unstimulated migration in non-treated, MANS treated or transfected (EGFP, MANS::EGFP or UMANS::EGFP) cells. These results confirmed that the MANS peptide, whether delivered by a cell permeable peptide approach or by epigenetic expression, significantly inhibits fibroblast migration. This data further demonstrates that myristoylation is not required for the ability of the MANS peptide to inhibit fibroblast migration and that other aspects of the MANS peptide, related specifically to the amino acid sequence, are involved in regulating MARCKS function related to fibroblast migration.

## Discussion

Herein, a role for MARCKS in regulation of NIH-3T3 fibroblast migration was demonstrated. Treatment with the MANS peptide, a myristoylated peptide corresponding to the first 24-amino acids of MARCKS that has been shown to inhibit MARCKS function, decreased migration of NIH-3T3 cells in scratch-wounding, PDGF-BB transmembrane chemotaxis assays and live-imaging chemotaxis assays. This finding is in accordance with previous studies from this laboratory demonstrating a role for MARCKS in migration of neutrophils [16], macrophages [17] and stem cells [18]. Given these results, we hypothesize that the amino-terminus of MARCKS is required for regulating MARCKS function during fibroblast migration, given that either the MANS peptide or epigenetically expressed MANS similarly attenuated NIH-3T3 migration. Further experiments are underway in our laboratory is necessary to address this hypothesis.

To begin to address the structural features of the MANS peptide involved in the mechanism by which the peptide inhibits fibroblast migration, we utilized a genetic structure-function approach to express MANS and unmyristoylated MANS (UMANS) C-terminal EGFP fusion proteins in NIH-3T3 cells. Similar expression of EGFP, MANS::EGFP and UMANS::EGFP was observed in transfected cells, as measured by EGFP expression. Further, equal expression of MARCKS was also observed in non-transfected cells and cell transfected with either EGFP, MANS::EGFP or UMANS::EGFP. It should be noted that in some instances, transformation of 3T3 cells results in decreased expression of MARCKS relative to non-transformed cells due to transcriptional down-regulation [40]–[42].

Previously, we demonstrated that isolated neutrophils are permeable to both the MANS and RNS peptides, presumably because of the amino-terminal myristoyl moiety. Further, subcellular fractionation studies demonstrated that MANS peptide displaces MARCKS from cell membranes in untreated neutrophils, whereas the RNS peptide allows for MARCKS to remain localized to the membrane [16]. Similarly, MANS peptide treatment resulted in displaced MARCKS binding from mucin granules in human bronchial epithelial cells, which correlated with decreased mucin secretion in asthma models [43]. Herein, we demonstrate that MANS::EGFP fusion proteins are preferentially targeted to the membrane fraction of NIH-3T3 fibroblasts, with some cytosolic localization. Conversely, EGFP and UMANS::EGFP are primarily localized in the cytosol, with some localization in the membrane fraction. It should be noted that the exact cellular membrane that MANS::EGFP and UMANS::EGFP localize to and how this affects MARCKS membrane localization have not been determined. However, we hypothesize that neither the MANS::EGFP nor UMANS::EGFP constructs are being retained in the endoplasmic reticulum or Golgi apparatus since we observed similar levels of attenuated cell migration in MANS peptide treated cells as cells that were transfected with MANS::EGFP or UMANS::EGFP (Figure 8). Taken together, these results suggest that the MANS peptide localizes to cell membranes and supports our previous hypothesis that MANS acts by competing with MARCKS for membrane binding sites within cells. Interestingly, the results also suggest that other aspect(s) of the MANS peptide besides the myristic acid moiety may be involved in tethering it to the plasma membrane as we observed low expression levels of UMANS::EGFP in membrane fractions.

Results of previous studies demonstrated that β_2_-integrin mediated neutrophil adhesion is regulated by MARCKS function although MARCKS does not affect expression of β_2_-integrins on neutrophils [16]. In the present study, a role for MARCKS in regulating fibroblast migration on both fibronectin and collagen substrates is demonstrated. Integrins that recognize collagen include α_1_β_1_ and α_2_β_1_ [44] while α_4_β_1_ and α_5_β_1_ are integrins that recognize fibronectin [45], [46]. NIH-3T3 cells express both fibronectin and collagen integrins [45], [47] and the results reported here suggest that MANS peptide mediated attenuation of NIH-3T3 migration occurs independent of the specific integrins that mediate adhesion to the substratum.

Herein, we also demonstrate that MANS peptide treatment does not alter the proliferation of NIH-3T3 fibroblasts as there was no difference in cell proliferation when NIH-3T3 fibroblasts were not treated or treated with MANS, RNS, PBS (VC) or wortmannin (Figure 2). This confirms that the decreased migration observed in the scratch-wounding assay (Figure 1) is due to altered cell migration and not cell proliferation. It should be noted that studies have revealed that MARCKS negatively regulates fibroblast proliferation as quiescent Swiss 3T3 cells express high levels of MARCKS mRNA and protein that are down-regulated upon cell proliferation [48]; similar observations have also been observed in other cell types [49], [50]. As shown in Figure 5, MANS peptide treatment does not alter MARCKS expression as equal MARCKS protein was observed in non-treated cells or cells treated with MANS, RNS or PBS (VC). Further, siRNA knockdown of MARCKS did not alter cell proliferation in comparison to non-treated or control siRNA transfected cells and MANS peptide treatment did not alter these observations (data not shown). Thus, while MANS may inhibit MARCKS function in regards to cell migration, MANS peptide treat does not alter MARCKS expression or cell proliferation within the parameters of an 18-hour scratch-wounding experiment. Further experimentation, which does not fall within the scope of this study, is needed to determine if MANS peptide treatment alters the proliferation of NIH-3T3 fibroblasts in experiments lasting longer than 18 hours.

As previously stated, we have demonstrated that MANS peptide treatment of neutrophils, macrophages and mesenchymal stem cells results in decreased chemotaxis [16]–[18]. Additionally, MARCKS phosphorylation and perinuclear translocation is observed in microglial cells stimulated by the chemoattractant amyloid beta protein, suggesting a role for MARCKS in microglial cell chemotaxis [51]–[53]. Further, modifications of MARCKS protein expression in hHSCs results in altered PDGF-BB chemotaxis [10]. Herein, we used a Boyden chamber and live-cell imaging approach [32] to show that MANS peptide treatment interferes with directional migration of fibroblasts. In the live-cell imaging experiments, MANS peptide treated cells did not exhibit high fidelity of chemotaxis and had a lesser degree of displacement from their original starting position in comparison to RNS treated cells. This demonstrates that MARCKS functions downstream of the chemokine receptor, possibly assisting in remodeling the actin cytoskeleton and/or relaying signals from the chemokine receptor to integrins that are essential for cell migration.

To address if MANS peptide treatment or decreased protein expression of MARCKS results in similar levels of attenuated migration, we knocked down the expression of MARCKS by siRNA treatment (Figure 6). Using a scratch-wounding assay, we observed similar levels of migration in non-treated, control siRNA or MARCKS A or B siRNA treated cells. Interestingly, Rombouts, *et al.* observed that increased PDGF-BB induced chemotaxis occurred in human hepatic stellate cells (hHSCs) that were transfected with a siRNA targeting MARCKS, while decreased chemotaxis was observed in hHSCs overexpressing MARCKS [10]. Further, alterations of human endothelial cell migration were not observed in cells transfected with a MARCKS siRNA [15]. Thus, our data supports those of others and demonstrates that MARCKS expression is not essential for migration of NIH-3T3 fibroblasts. Our data also suggests that an alternative, unidentified mediator compensates for MARCKS function in fibroblasts expressing decreased MARCKS levels. MARCKS is likely preferred over this unidentified mediator during the cell migration process as cells with inhibited MARCKS function due to MANS peptide treatment have ameliorated cell migration while cells with decreased MARCKS expression are not affected by MANS peptide treatment (Figure 6). It should be noted, however, that MARCKS knockdown in human vascular smooth muscle cells does result in ameliorated cell migration [15], suggesting that the mechanism by which MARCKS regulates cell migration may be cell type specific.

The results in Figure 6 also demonstrate that the MANS peptide specifically inhibits the function of MARCKS during fibroblast migration as MANS peptide treatment does not alter the migration of cells with decreased MARCKS protein expression. As previously stated, our lab has shown that the MANS peptide displaces MARCKS from the plasma membrane of neutrophils as well as from mucin granules of human bronchial epithelial cells [16], [43], resulting in decreased cell migration and mucin secretion, respectively. While these studies demonstrate that MANS specifically interferes with MARCKS membrane localization and presumably function, these studies do not rule out the possibility that MANS peptide treatment may also have off-target effects. Given that we did not observe any difference in cell migration in MARCKS A or B siRNA transfected cells that were also treated with the MANS peptide, we can conclude that the MANS peptide specifically interferes with MARCKS function and does not have any off-target effects.

The exact mechanism by which the MANS peptide inhibits MARCKS function has yet to be determined, although it is hypothesized that MANS may compete with the ability of MARCKS to interact with other mediators involved in the cell migration process. At the present time, it is unclear as to the specific MARCKS interactions that the MANS peptide may interfere with, although a possibility could be MARCKS and phosphatidylinositol 4, 5-bisphosphate (PIP_2_) interactions. The unphosphorylated effector domain of MARCKS binds to PIP_2_-rich regions in the plasma membrane, thus clustering and sequestering PIP_2_ molecules. This MARCKS mediated sequestration of PIP_2_ has been shown to inhibit phospholipase C-gamma (PLC-gamma) mediated hydrolysis of PIP_2_. Upon chemoattractant stimulation, MARCKS becomes phosphorylated or bound by CaM and dissociates from the plasma membrane, releasing its sequestration of PIP_2_ and thereby allowing PLC-gamma to hydrolyze PIP_2_ [54]–[56]. Actin cytoskeletal dynamics are regulated by the concentration of free PIP_2_, as the concentration of PIP_2_ signals for anchoring or releasing the actin cytoskeleton from the plasma membrane [54], [57], [58]. Thus, PIP_2_ acts to tightly regulate the process of cell migration by coordinated attachment and release of the actin cytoskeleton from the plasma membrane. PIP_2_ is also involved in talin activation, with talin activating and regulating integrin function, which is essential to cell migraton [59]–[61]. As described above, MANS peptide treatment displaces MARCKS from the plasma membrane in non-stimulated neutrophils [16] as well as from mucin granules in human bronchial epithelial cells [43], demonstrating that MANS competitively inhibits MARCKS binding to the plasma membrane. Given these observations, we hypothesize that the mechanism by which MANS peptide interferes with directed cell migration could be through lack of MARCKS mediated PIP_2_ sequestration, resulting in the constant availability of PIP_2_ to be hydrolyzed by PLC-gamma. Additional studies are underway in our laboratory to address this hypothesis.

Previous work has demonstrated that unmyristoylated MARCKS preferentially localizes to the cytosol, with some localization to the membrane, and it is thought that low-level membrane localization of unmyristoylated MARCKS is due to weak electrostatic interactions between the effector domain of MARCKS and the plasma membrane [3], [13], [62]. Interestingly, myoblasts transfected with unmyristoylated MARCKS were still capable of adhesion and spreading on a fibronectin substrate while MARCKS effector domain deletion mutants were not, suggesting that myristoylation of MARCKS is not essential to it’s function [13]. Given this, we can be fairly certain that unmyristoylated MARCKS would likely not affect cell migration, as adhesion and spreading are key steps to the process of cell motility.

Results of the studies reported here indicate that myristoylation of the MANS peptide itself is not required for MANS peptide-mediated inhibition of MARCKS regulated fibroblast migration. Epigenetically expressed unmyristoylated MANS (UMANS::EGFP), which was localized to both the membrane and cytosol, was capable of inhibiting cell migration in a similar manner to MANS peptide treatment or epigenetic expression of MANS::EGFP. These findings demonstrate that myristoylation of the MANS peptide is not essential for inhibition of cell migration. This data further suggests that membrane localization of the MANS peptide is not essential to disrupting MARCKS function and other aspects of the MANS peptide may be involved in regulating MARCKS function. There are specific amino acids within the MANS peptide (and the amino-terminus of MARCKS) that could potentially be involved in regulating MARCKS function. Candidate amino acids within the amino-terminus of MARCKS that may be involved in regulating cell migration are Lys^6^ and Thr^7^, as it is known that proteolytic cleavage occurs at this site by an unidentified protease [63]. Calpain, a potential candidate for the unidentified protease [64], [65], is a calcium-activated protease that is localized to the leading edge of polarized neutrophils and is involved in pseudopod formation and chemotaxis [66], [67]. It is also involved in lymphocyte function-associated antigen-1 (LFA-1) mediated T-lymphocyte adhesion as well as focal adhesion formation in bovine aortic endothelial cells [68], [69]. Calpain is known to cleave MARCKS in myoblasts, resulting in a 55 kDa fragment [64] and inhibition of calpain activity resulted in decreased myoblast migration associated with an accumulation of membrane bound MARCKS [64], [70]–[72]. Further, adenosine triphosphate (ATP)-mediated activation of calpain results in amino-terminal MARCKS cleavage products in virally transformed human bronchial epithelial cells (HBE-1) [73]. Thus, we hypothesize that generation of the six amino acid fragment by proteolytic cleavage of MARCKS between Lys^6^ and Thr^7^ may be involved in regulating cell migration, with further experimentation needed to confirm this hypothesis.

PDGF-BB, a known mitogen and chemoattractant for fibroblasts, signals through a receptor tyrosine kinase, PDGFR. Signaling through PDGFR increases intracellular Ca^2+^ concentrations and activates PKC, both of which mediate MARCKS function [31], [74]. In Swiss 3T3 cells, PDGF-BB stimulation results in phosphorylation of MARCKS and subsequent membrane to cytosol translocation [35]–[37]. MARCKS can be phosphorylated by PKCα, PKCε and PKCθ isoforms in NIH-3T3 cells [75] and PDGF-BB is known to activate PKC-α in various fibroblast lines [76], [77]. PDGF-BB mediated MARCKS phosphorylation is dependent on both PKC-α and –ε during the migration of hHSCs [10]. Thus, it is likely that PKCα is involved in PDGF-BB induced MARCKS phosphorylation in the studies reported here, although identification of the exact PKC isoform(s) involved was not performed. Interestingly, MANS peptide treatment did not alter PDGF-BB mediated phosphorylation of MARCKS in adherent NIH-3T3 fibroblasts (Figure 5), demonstrating that MANS peptide mediated attenuation of fibroblast migration is not due to abnormalities in MARCKS phosphorylation.

Additionally, there were no observed abnormalities in PI3K activity in MANS or RNS treated cells, as measured by an EGFP-AktPH biosensor (Figure 4) or phospho-Akt Western Blot analysis (Figure 5). PDGF-BB signaling in fibroblasts results in PI3K localization to the polarized leading edge, and inhibition of PI3K activity results in attenuated cell migration [28], [32]. Given that abnormal PI3K signaling in MANS treated cells was not observed, these results indicate that the MANS peptide-attenuated cell migration occurs independently of PI3K activation. However, PI3K signaling is known to be involved in PKC activation and subsequent MARCKS phosphorylation [78], [79], so it appears that while PI3K may affect MARCKS function, MARCKS does not appear to affect PI3K activity.

In summary, these studies have indicated an important role for MARCKS in regulating NIH-3T3 fibroblast migration, although MARCKS expression is not essential to the process. Further, treatment of cells with the MANS peptide, which specifically inhibits MARCKS function, results in ameliorated cell migration. MANS peptide mediated inhibition of cell migration occurs regardless of amino-terminal myristoylation. Given that the MANS peptide has also been shown to inhibit neutrophil, macrophages and mesenchymal stem cell migration [16]–[18] the results reported here provide further evidence for the concept that inhibiting MARCKS function may be a valuable novel therapeutic approach for various diseases associated with exacerbated cell migration, such as inflammation, injury/repair and metastatic disease.

## Materials and Methods

### Reagents and Cell Culture

MANS and RNS peptides were synthesized as previously described [39] and resuspended in sterile PBS. Wortmannin was obtained from Sigma (St. Louis, MO) and a stock solution was made in DMSO (Sigma). PDGF-BB and fatty acid free bovine serum albumin (BSA) were purchased from Sigma and were resuspended in sterile water or PBS, respectively. Type II rat-tail collagen and fibronectin (Sigma) were resuspended in 0.1% acetic acid (v/v) or sterile water, respectively.

NIH-3T3 fibroblasts (ATCC, Manassas, VA) were maintained in Dulbecco’s Modified Eagle Medium (DMEM; Mediatech, Manassas, VA) supplemented with 10% fetal bovine serum (FBS; Gemini Bio-Products, West Sacramento, CA) and 0.2% Penicillin (10,000 U/ml) Streptomycin (10,000 µg/ml) solution (Gemini Bio-Products).

### Recombinant Plasmids and Transfections

MANS and UMANS inserts were PCR amplified from pCDNA4/TO wt MARCKS plasmid with glycine (GGT) to alanine (GCT) point mutation in the second amino acid position for UMANS. MANS and UMANS were cloned into the *EcoRI* and *BamHI* restriction sites of pEGFP-N1 (Clonetech, Mountain View, CA); colonies were screened by colony PCR using pEGFP-N1 sequencing primers and positive colonies were sequenced (MWG, Huntsville, AL). Qiagnen’s EndoFree Maxi Kit (Qiagen, Valencia, CA) was used to prepare purified plasmid that was endotoxin free. NIH-3T3 fibroblasts were transfected by nucleofection using the Amaxa® Cell Line Nucleofector Kit® R (Lonza, Basel, Switzerland) following manufacturer’s protocol with 10 µg of plasmid per reaction. Transfected cells were plated in 6-well tissue culture plates and the expression of EGFP fusion proteins was determined by fluorescent microscopy or western blot analysis 18 hours after transfection; transfected cells were used for cell migration analysis within 24 hours.

siRNAs (Origene Technology, Rockville, MD) targeting the following sequences were used: 5′-GGAGTTCATGRAAACCATAGGAACT-3′ (MARCKS A), 5′-GGAATGTAACGTTGCTTACAAGCAT-3′ (MARCKS B). Cells (1×10^5^) in a 6-well plate were transfected using Lipofectamine RNAiMAX reagent (Invitrogen) with 10 nM of MARCKS A, B or control siRNA and knockdown was confirmed by Western blot 48 after transfection.

### Scratch Wounding Assay

Fibroblast scratch assays were performed as described [80]. Briefly, sterile 22-mm coverslips (Fisher Scientific, Pittsburgh, PA) were coated with 10 µg/mL fibronectin or collagen in sterile 6-well tissue culture plates for two hours at room temperature. Coated coverslips were washed in sterile PBS and NIH-3T3 fibroblasts were seeded in complete media and cultured until confluent. For scratch assays with transfected cells, two nucleofection reactions per scratch were performed (1×10^6^ cells/nucleofection or 2×10^6^ cells/scratch assay) with scratch assays starting 18–24 hours after transfection. For scratch assays on siRNA knockdown cells, one siRNA reaction per scratch was performed, with scratch assays occurring 48 hours after transfection. Two parallel scratches that were consistent in width were made in the monolayer using a standard sterile 200 µL pipette tip. The coverslips were washed with sterile PBS and replaced with DMEM containing 2% FBS and antibiotics. In some experiments, media was supplemented with MANS, RNS, sterile PBS (VC) or 100 nM wortmannin. The T = 0 coverslip was immediately removed and processed prior to incubating the remainder of the plate for 18 hours at 37°C, 5% CO_2_. Coverslips were processed by fixing in 10% neutral buffered formalin solution (Fisher) and stained with Harris Hematoxylin or Diff-Quick following standard procedures. Coverslips were mounted onto microscope slides and an ocular micrometer was used to measure the wound distance at ten random locations along the scratch under at 40×. Wound closure distance for each sample was determined by subtracting the average wound closure for each sample from the average initial T = 0 wound distance with data represented as average percent wound closure ± standard error of the mean (SEM). Photographs of the scratches were obtained using a Nikon AZ100 microscope (Nikon, Melville, NY) under bright field conditions.

### CFSE Proliferation Assay

NIH-3T3 fibroblasts were loaded with 2 µM CFSE (eBioscience, San Diego, CA) and plated (5×10^5^ cells/well) on fibronectin (10 µg/mL) coated 6-well plates in complete media (DMEM with 10% FBS and antibiotics). Cells were allowed to adhere to the plate for 2 hours at 37°C, 5% CO_2_ and then washed twice with sterile PBS. DMEM with 2% FBS and antibiotics was added to each well and cells were treated with either 50 µM MANS, 50 µM RNS, PBS (VC) or 100 nM wortmannin before incubating for 18 hours at 37°C, 5% CO_2._ Cells were then harvested and fixed in 1% paraformaldehyde in PBS and cell proliferation was evaluated by flow cytometry using an Accuri C6 flow cytometer (30,000 total events collected) with data analysis performed using FloJo software.

### Transmembrane Chemotaxis Assay

Fibroblast chemotaxis assays were performed as described [81]. Briefly, transwell inserts (8 µm pore size, 6.6 mm diameter; Corning, Corning, NY) were coated with 10 µg/mL fibronectin for two hours at room temperature and washed in PBS. Transfected or non-transfected NIH-3T3 cells were washed and resuspended in sterile serum-free DMEM at a concentration of 5×10^5^ cells/ml. In some experiments, cells were pretreated with 50 µM MANS, 50 µM RNS, sterile PBS (VC) or 100 nM wortmannin for 30 minutes at 37°C. Chemotaxis buffer consisted of serum free DMEM containing 1 mg/mL fatty acid free BSA; 1 nM PDGF-BB or VC (sterile water) was added to the chemotaxis buffer prior to addition to a 24-well plate with fibronectin coated transwells placed on top. NIH-3T3 cells (100 µL or 5×10^4^ cells) were placed in the top chamber of each transwell and the plate was incubated for 4 hours at 37°C, 5% CO_2._ Cells on the upper part of the filter were dislodged with a sterile cotton swab and rinsed with sterile PBS. Filters were fixed in 10% neutral buffered formalin solution and stained with harris hematoxylin or Diff-Quick prior to removing the filters from the transwell and mounting on glass microscope slides. The number of cells on the bottom of the filter was counted in 10 randomly selected high-powered fields (400×) of a light microscope.

### Western Blotting and PDGF Stimulation

For PDGF stimulation of adherent cells, NIH-3T3 fibroblasts were seeded and grown to 90% confluence on 6-well plates that were coated with 10 µg/mL fibronectin. Cells were serum starved for 4 hours in serum-free DMEM plus antibiotics and 1 mg/mL fatty acid free BSA. In some experiments, cells were pretreated with 50 µM MANS, RNS or VC (PBS) for 30 minutes at 37°C and cells were stimulated with PDGF-BB for the indicated time and plates were immediately placed on ice. Cells were washed with ice cold sterile PBS prior to the addition of RIPA buffer containing protease inhibitors (1% NP-40, 0.5% sodium deoxycholate, 0.1% sodium dodecyl sulfate (SDS), 5 mM sodium pyrophosphate and 50 mM sodium fluoride1 mM phenylmethanesulphonylfluoride (PMSF) and 1∶100 dilution of Sigma protease inhibitor cocktail) and were removed from the plate by scraping. Lysates were prepared by standard procedure and protein concentrations were determined by the BCA assay (Pierce, Rockford, IL). Boiled samples, diluted in 4× lithium dodecyl sulfate (LDS) buffer, were separated by 4–12% SDS-PAGE. Phospho-MARCKS (Ser 152/156), phospho-AKT1 (Ser 473), total AKT1 and beta-actin antibodies were purchased from Cell Signaling Technology (Danvers MA) and total MARCKS (M-20) was purchased from Santa Cruz Biotechnology (Santa Cruz, CA). Western Blots were developed by ECL enhanced chemiluminescence (Thermo Scientific, Rockford, IL) and exposed to radiographic film.

### Subcellular Fractionation

Subcellular fractionation was performed using the Subcellular Protein Fractionation Kit (Thermo Scientific) 24 hours after transfection according to the manufacturer’s instruction. Equal protein concentrations were loaded onto a 4–12% SDS-PAGE and Western blots were performed with the following antibodies: EGFP polyclonal (Santa Cruz Biotechnology), p38 MAPK (α, β, γ isoforms; Cell Signaling) and beta-actin (Cell Signaling). Western Blots were developed by ECL enhanced chemiluminescence and exposed to radiographic film.

### Live-cell Fluorescence Microscopy and Image Analysis

Stable expression of the 3' phosphoinositide-specific biosensor construct EGFP-AktPH in NIH 3T3 cells was established by retroviral infection as described previously [28]. These cells were plated on glass coated with human plasma fibronectin (10 mg/mL coating concentration, obtained from BD Biosciences (San Jose, CA)). The imaging buffer was 20 mM HEPES pH 7.4, 125 mM NaCl, 5 mM KCl, 1.5 mM MgCl_2_, 1.5 mM CaCl_2_, 10 mM glucose, and 2 mg/mL fatty acid-free bovine serum albumin, supplemented with 1% (v/v) fetal bovine serum. Localization of PI3K signaling was monitored by total internal reflection fluorescence (TIRF) using a prism-based microscope described in detail previously [28], [34]. Chemotaxis experiments using alginate microspheres (a kind gift from Darrell Irvine, MIT) were carried out essentially as described [32], except that RNS or MANS peptide was added to the cells 30 minutes prior to the addition of the microspheres. Calculations of each cell’s centroid coordinates and the angle of its movement relative to the estimated PDGF gradient were performed as described [32].

### Statistical Analysis

Statistical analysis was performed by Sigma Stat Software (Systat Software, Inc, Chicago, IL) using Student’s t-test with P-values less than or equal to 0.05 considered statistically significant.

## References

[1] James G, Olson EN (1989) Myristoylation, phosphorylation, and subcellular distribution of the 80-kDa protein kinase C substrate in BC3H1 myocytes. J Biol Chem 264: 20928–20933. 2592358

[2] Denisov G, Wanaski S, Luan P, Glaser M, McLaughlin S (1998) Binding of basic peptides to membranes produces lateral domains enriched in the acidic lipids phosphatidylserine and phosphatidylinositol 4,5-bisphosphate: An electrostatic model and experimental results. Biophys J 74: 731–744 10.1016/S0006-3495(98)73998-0. 9533686 10.1016/S0006-3495(98)73998-0 PMC1302554

[3] Swierczynski SL, Blackshear PJ (1995) Membrane association of the myristoylated alanine-rich C kinase substrate (MARCKS) protein. mutational analysis provides evidence for complex interactions. J Biol Chem 270: 13436–13445. 7768946 10.1074/jbc.270.22.13436

[4] Yarmola EG, Edison AS, Lenox RH, Bubb MR (2001) Actin filament cross-linking by MARCKS: Characterization of two actin-binding sites within the phosphorylation site domain. J Biol Chem 276: 22351–22358 10.1074/jbc.M101457200. 11294839 10.1074/jbc.M101457200

[5] Hartwig JH, Thelen M, Rosen A, Janmey PA, Nairn AC, et al (1992) MARCKS is an actin filament crosslinking protein regulated by protein kinase C and calcium-calmodulin. Nature 356: 618–622 10.1038/356618a0. 1560845 10.1038/356618a0

[6] Song JC, Hrnjez BJ, Farokhzad OC, Matthews JB (1999) PKC-epsilon regulates basolateral endocytosis in human T84 intestinal epithelia: Role of F-actin and MARCKS. Am J Physiol 277: C1239–49. 10600776 10.1152/ajpcell.1999.277.6.C1239

[7] Satoh K, Matsuki-Fukushima M, Qi B, Guo MY, Narita T, et al (2009) Phosphorylation of myristoylated alanine-rich C kinase substrate is involved in the cAMP-dependent amylase release in parotid acinar cells. Am J Physiol Gastrointest Liver Physiol 296: G1382–90 10.1152/ajpgi.90536.2008. 19372103 10.1152/ajpgi.90536.2008

[8] Allen LH, Aderem A (1995) A role for MARCKS, the alpha isozyme of protein kinase C and myosin I in zymosan phagocytosis by macrophages. J Exp Med 182: 829–840. 7650489 10.1084/jem.182.3.829 PMC2192156

[9] Carballo E, Pitterle DM, Stumpo DJ, Sperling RT, Blackshear PJ (1999) Phagocytic and macropinocytic activity in MARCKS-deficient macrophages and fibroblasts. Am J Physiol 277: C163–73. 10409119 10.1152/ajpcell.1999.277.1.C163

[10] Rombouts K, Lottini B, Caligiuri A, Liotta F, Mello T, et al (2008) MARCKS is a downstream effector in platelet-derived growth factor-induced cell motility in activated human hepatic stellate cells. Exp Cell Res 314: 1444–1454 10.1016/j.yexcr.2008.01.029. 18329017 10.1016/j.yexcr.2008.01.029

[11] Techasen A, Loilome W, Namwat N, Takahashi E, Sugihara E, et al (2010) Myristoylated alanine-rich C kinase substrate phosphorylation promotes cholangiocarcinoma cell migration and metastasis via the protein kinase C-dependent pathway. Cancer Sci 101: 658–665 10.1111/j.1349-7006.2009.01427.x. 20047593 10.1111/j.1349-7006.2009.01427.x PMC11158558

[12] Myat MM, Anderson S, Allen LA, Aderem A (1997) MARCKS regulates membrane ruffling and cell spreading. Curr Biol 7: 611–614. 9259558 10.1016/s0960-9822(06)00262-4

[13] Disatnik MH, Boutet SC, Pacio W, Chan AY, Ross LB, et al (2004) The bi-directional translocation of MARCKS between membrane and cytosol regulates integrin-mediated muscle cell spreading. J Cell Sci 117: 4469–4479 10.1242/jcs.01309. 15316066 10.1242/jcs.01309

[14] Spizz G, Blackshear PJ (2001) Overexpression of the myristoylated alanine-rich C-kinase substrate inhibits cell adhesion to extracellular matrix components. J Biol Chem 276: 32264–32273 10.1074/jbc.M103960200. 11413143 10.1074/jbc.M103960200

[15] Monahan TS, Andersen ND, Martin MC, Malek JY, Shrikhande GV, et al (2009) MARCKS silencing differentially affects human vascular smooth muscle and endothelial cell phenotypes to inhibit neointimal hyperplasia in saphenous vein. FASEB J 23: 557–564 10.1096/fj.08-114173. 18940893 10.1096/fj.08-114173 PMC2630782

[16] Eckert RE, Neuder LE, Park J, Adler KB, Jones SL (2010) Myristoylated alanine-rich C-kinase substrate (MARCKS) protein regulation of human neutrophil migration. Am J Respir Cell Mol Biol 42: 586–594 10.1165/rcmb.2008-0394OC. 19574534 10.1165/rcmb.2008-0394OC PMC2874444

[17] Green TD, Park J, Yin Q, Fang S, Crews AL, et al (2012) Directed migration of mouse macrophages in vitro involves myristoylated alanine-rich C-kinase substrate (MARCKS) protein. J Leukoc Biol 92: 633–639 10.1189/jlb.1211604; 10.1189/jlb.1211604. 22623357 10.1189/jlb.1211604 PMC3427602

[18] Miller JD, Lankford SM, Adler KB, Brody AR (2010) Mesenchymal stem cells require MARCKS protein for directed chemotaxis in vitro. Am J Respir Cell Mol Biol 43: 253–258 10.1165/rcmb.2010-0015RC. 20224071 10.1165/rcmb.2010-0015RC PMC3159077

[19] Stensman H, Larsson C (2008) Protein kinase cepsilon is important for migration of neuroblastoma cells. BMC Cancer 8: 365 10.1186/1471-2407-8-365. 19077250 10.1186/1471-2407-8-365 PMC2615448

[20] Micallef J, Taccone M, Mukherjee J, Croul S, Busby J, et al (2009) Epidermal growth factor receptor variant III-induced glioma invasion is mediated through myristoylated alanine-rich protein kinase C substrate overexpression. Cancer Res 69: 7548–7556 10.1158/0008-5472.CAN-08-4783. 19773446 10.1158/0008-5472.CAN-08-4783

[21] Disatnik MH, Boutet SC, Lee CH, Mochly-Rosen D, Rando TA (2002) Sequential activation of individual PKC isozymes in integrin-mediated muscle cell spreading: A role for MARCKS in an integrin signaling pathway. J Cell Sci 115: 2151–2163. 11973356 10.1242/jcs.115.10.2151

[22] Aderem A (1992) The MARCKS brothers: A family of protein kinase C substrates. Cell 71: 713–716. 1423627 10.1016/0092-8674(92)90546-o

[23] Seykora JT, Myat MM, Allen LA, Ravetch JV, Aderem A (1996) Molecular determinants of the myristoyl-electrostatic switch of MARCKS. J Biol Chem 271: 18797–18802. 8702537 10.1074/jbc.271.31.18797

[24] Blackshear PJ, Verghese GM, Johnson JD, Haupt DM, Stumpo DJ (1992) Characteristics of the F52 protein, a MARCKS homologue. J Biol Chem 267: 13540–13546. 1618855

[25] Li J, Aderem A (1992) MacMARCKS, a novel member of the MARCKS family of protein kinase C substrates. Cell 70: 791–801. 1516135 10.1016/0092-8674(92)90312-z

[26] Umekage T, Kato K (1991) A mouse brain cDNA encodes a novel protein with the protein kinase C phosphorylation site domain common to MARCKS. FEBS Lett 286: 147–151. 1864362 10.1016/0014-5793(91)80961-2

[27] Chun KR, Bae EM, Kim JK, Suk K, Lee WH (2009) Suppression of the lipopolysaccharide-induced expression of MARCKS-related protein (MRP) affects transmigration in activated RAW264.7 cells. Cell Immunol 256: 92–98 10.1016/j.cellimm.2009.01.011. 19246034 10.1016/j.cellimm.2009.01.011

[28] Weiger MC, Wang CC, Krajcovic M, Melvin AT, Rhoden JJ, et al (2009) Spontaneous phosphoinositide 3-kinase signaling dynamics drive spreading and random migration of fibroblasts. J Cell Sci 122: 313–323 10.1242/jcs.037564. 19126672 10.1242/jcs.037564 PMC2724728

[29] Bell PB Jr (1977) Locomotory behavior, contact inhibition and pattern formation of 3T3 and polyoma virus-transformed 3T3 cells in culture. J Cell Biol 74: 963–982. 198414 10.1083/jcb.74.3.963 PMC2110095

[30] Bonner JC (2004) Regulation of PDGF and its receptors in fibrotic diseases. Cytokine Growth Factor Rev 15: 255–273 10.1016/j.cytogfr.2004.03.006. 15207816 10.1016/j.cytogfr.2004.03.006

[31] Andrae J, Gallini R, Betsholtz C (2008) Role of platelet-derived growth factors in physiology and medicine. Genes Dev 22: 1276–1312 10.1101/gad.1653708. 18483217 10.1101/gad.1653708 PMC2732412

[32] Melvin AT, Welf ES, Wang Y, Irvine DJ, Haugh JM (2011) In chemotaxing fibroblasts, both high-fidelity and weakly biased cell movements track the localization of PI3K signaling. Biophys J 100: 1893–1901 10.1016/j.bpj.2011.02.047. 21504725 10.1016/j.bpj.2011.02.047 PMC3077704

[33] Schneider IC, Haugh JM (2006) Mechanisms of gradient sensing and chemotaxis: Conserved pathways, diverse regulation. Cell Cycle 5: 1130–1134. 16760661 10.4161/cc.5.11.2770

[34] Schneider IC, Haugh JM (2005) Quantitative elucidation of a distinct spatial gradient-sensing mechanism in fibroblasts. J Cell Biol 171: 883–892 10.1083/jcb.200509028. 16314431 10.1083/jcb.200509028 PMC2171296

[35] Rozengurt E, Rodriguez-Pena M, Smith KA (1983) Phorbol esters, phospholipase C, and growth factors rapidly stimulate the phosphorylation of a mr 80,000 protein in intact quiescent 3T3 cells. Proc Natl Acad Sci U S A 80: 7244–7248. 6316349 10.1073/pnas.80.23.7244 PMC390031

[36] Isacke CM, Meisenhelder J, Brown KD, Gould KL, Gould SJ, et al (1986) Early phosphorylation events following the treatment of swiss 3T3 cells with bombesin and the mammalian bombesin-related peptide, gastrin-releasing peptide. EMBO J 5: 2889–2898. 2431903 10.1002/j.1460-2075.1986.tb04584.x PMC1167239

[37] Herget T, Rozengurt E (1994) Bombesin, endothelin and platelet-derived growth factor induce rapid translocation of the myristoylated alanine-rich C-kinase substrate in swiss 3T3 cells. Eur J Biochem 225: 539–548. 7957168 10.1111/j.1432-1033.1994.00539.x

[38] Arbuzova A, Schmitz AA, Vergeres G (2002) Cross-talk unfolded: MARCKS proteins. Biochem J 362: 1–12. 11829734 10.1042/0264-6021:3620001 PMC1222354

[39] Li Y, Martin LD, Spizz G, Adler KB (2001) MARCKS protein is a key molecule regulating mucin secretion by human airway epithelial cells in vitro. J Biol Chem 276: 40982–40990 10.1074/jbc.M105614200. 11533058 10.1074/jbc.M105614200

[40] Joseph CK, Qureshi SA, Wallace DJ, Foster DA (1992) MARCKS protein is transcriptionally down-regulated in v-src-transformed BALB/c 3T3 cells. J Biol Chem 267: 1327–1330. 1370466

[41] Otsuka M, Yang HC (1991) Decreased expression of the myristoylated alanine-rich C kinase substrate in transformed BALB/C 3T3 mouse fibroblasts. Biochem Biophys Res Commun 178: 494–500. 1713448 10.1016/0006-291x(91)90134-s

[42] Reed JC, Rapp U, Cuddy MP (1991) Transformed 3T3 cells have reduced levels and altered subcellular distribution of the major PKC substrate protein MARCKS. Cell Signal 3: 569–576. 1838487 10.1016/0898-6568(91)90033-q

[43] Singer M, Martin LD, Vargaftig BB, Park J, Gruber AD, et al (2004) A MARCKS-related peptide blocks mucus hypersecretion in a mouse model of asthma. Nat Med 10: 193–196 10.1038/nm983. 14716307 10.1038/nm983

[44] McBrien NA, Metlapally R, Jobling AI, Gentle A (2006) Expression of collagen-binding integrin receptors in the mammalian sclera and their regulation during the development of myopia. Invest Ophthalmol Vis Sci 47: 4674–4682 10.1167/iovs.05-1150. 17065473 10.1167/iovs.05-1150

[45] Dalton SL, Marcantonio EE, Assoian RK (1992) Cell attachment controls fibronectin and alpha 5 beta 1 integrin levels in fibroblasts. implications for anchorage-dependent and -independent growth. J Biol Chem 267: 8186–8191. 1373721

[46] Barczyk M, Carracedo S, Gullberg D (2010) Integrins. Cell Tissue Res 339: 269–280 10.1007/s00441-009-0834-6. 19693543 10.1007/s00441-009-0834-6 PMC2784866

[47] Kato S, Ben TL, De Luca LM (1988) Phorbol esters enhance attachment of NIH/3T3 cells to laminin and type IV collagen substrates. Exp Cell Res 179: 31–41. 3169149 10.1016/0014-4827(88)90345-x

[48] Herget T, Brooks SF, Broad S, Rozengurt E (1993) Expression of the major protein kinase C substrate, the acidic 80-kilodalton myristoylated alanine-rich C kinase substrate, increases sharply when swiss 3T3 cells move out of cycle and enter G0. Proc Natl Acad Sci U S A 90: 2945–2949. 8464911 10.1073/pnas.90.7.2945 PMC46213

[49] Manenti S, Malecaze F, Chap H, Darbon JM (1998) Overexpression of the myristoylated alanine-rich C kinase substrate in human choroidal melanoma cells affects cell proliferation. Cancer Res 58: 1429–1434. 9537244

[50] Zhao Y, Neltner BS, Davis HW (2000) Role of MARCKS in regulating endothelial cell proliferation. Am J Physiol Cell Physiol 279: C1611–20. 11029309 10.1152/ajpcell.2000.279.5.C1611

[51] Nakai M, Hojo K, Taniguchi T, Terashima A, Kawamata T, et al (1998) PKC and tyrosine kinase involvement in amyloid beta (25–35)-induced chemotaxis of microglia. Neuroreport 9: 3467–3470. 9855300 10.1097/00001756-199810260-00024

[52] Nakai M, Hojo K, Yagi K, Saito N, Taniguchi T, et al (1999) Amyloid beta protein (25–35) phosphorylates MARCKS through tyrosine kinase-activated protein kinase C signaling pathway in microglia. J Neurochem 72: 1179–1186. 10037491 10.1046/j.1471-4159.1999.0721179.x

[53] Nakai M, Tanimukai S, Yagi K, Saito N, Taniguchi T, et al (2001) Amyloid beta protein activates PKC-delta and induces translocation of myristoylated alanine-rich C kinase substrate (MARCKS) in microglia. Neurochem Int 38: 593–600. 11290384 10.1016/s0197-0186(00)00126-1

[54] Glaser M, Wanaski S, Buser CA, Boguslavsky V, Rashidzada W, et al (1996) Myristoylated alanine-rich C kinase substrate (MARCKS) produces reversible inhibition of phospholipase C by sequestering phosphatidylinositol 4,5-bisphosphate in lateral domains. J Biol Chem 271: 26187–26193. 8824266 10.1074/jbc.271.42.26187

[55] Laux T, Fukami K, Thelen M, Golub T, Frey D, et al (2000) GAP43, MARCKS, and CAP23 modulate PI(4,5)P(2) at plasmalemmal rafts, and regulate cell cortex actin dynamics through a common mechanism. J Cell Biol 149: 1455–1472. 10871285 10.1083/jcb.149.7.1455 PMC2175130

[56] Sundaram M, Cook HW, Byers DM (2004) The MARCKS family of phospholipid binding proteins: Regulation of phospholipase D and other cellular components. Biochem Cell Biol 82: 191–200 10.1139/o03-087. 15052337 10.1139/o03-087

[57] McLaughlin S, Wang J, Gambhir A, Murray D (2002) PIP(2) and proteins: Interactions, organization, and information flow. Annu Rev Biophys Biomol Struct 31: 151–175 10.1146/annurev.biophys.31.082901.134259. 11988466 10.1146/annurev.biophys.31.082901.134259

[58] Sechi AS, Wehland J (2000) The actin cytoskeleton and plasma membrane connection: PtdIns(4,5)P(2) influences cytoskeletal protein activity at the plasma membrane. J Cell Sci 113 Pt 21: 3685–3695. 10.1242/jcs.113.21.3685 11034897

[59] Alon R, Shulman Z (2011) Chemokine triggered integrin activation and actin remodeling events guiding lymphocyte migration across vascular barriers. Exp Cell Res 317: 632–641 10.1016/j.yexcr.2010.12.007. 21376176 10.1016/j.yexcr.2010.12.007

[60] Goksoy E, Ma YQ, Wang X, Kong X, Perera D, et al (2008) Structural basis for the autoinhibition of talin in regulating integrin activation. Mol Cell 31: 124–133 10.1016/j.molcel.2008.06.011. 18614051 10.1016/j.molcel.2008.06.011 PMC2522368

[61] Saltel F, Mortier E, Hytonen VP, Jacquier MC, Zimmermann P, et al (2009) New PI(4,5)P2- and membrane proximal integrin-binding motifs in the talin head control beta3-integrin clustering. J Cell Biol 187: 715–731 10.1083/jcb.200908134. 19948488 10.1083/jcb.200908134 PMC2806581

[62] Ohmori S, Sakai N, Shirai Y, Yamamoto H, Miyamoto E, et al (2000) Importance of protein kinase C targeting for the phosphorylation of its substrate, myristoylated alanine-rich C-kinase substrate. J Biol Chem 275: 26449–26457 10.1074/jbc.M003588200. 10840037 10.1074/jbc.M003588200

[63] Braun T, McIlhinney RA, Vergeres G (2000) Myristoylation-dependent N-terminal cleavage of the myristoylated alanine-rich C kinase substrate (MARCKS) by cellular extracts. Biochimie 82: 705–715. 11018286 10.1016/s0300-9084(00)01154-8

[64] Dulong S, Goudenege S, Vuillier-Devillers K, Manenti S, Poussard S, et al (2004) Myristoylated alanine-rich C kinase substrate (MARCKS) is involved in myoblast fusion through its regulation by protein kinase calpha and calpain proteolytic cleavage. Biochem J 382: 1015–1023 10.1042/BJ20040347. 15239673 10.1042/BJ20040347 PMC1133979

[65] Tapp H, Al-Naggar IM, Yarmola EG, Harrison A, Shaw G, et al (2005) MARCKS is a natively unfolded protein with an inaccessible actin-binding site: Evidence for long-range intramolecular interactions. J Biol Chem 280: 9946–9956 10.1074/jbc.M414614200. 15640140 10.1074/jbc.M414614200

[66] Nuzzi PA, Senetar MA, Huttenlocher A (2007) Asymmetric localization of calpain 2 during neutrophil chemotaxis. Mol Biol Cell 18: 795–805 10.1091/mbc.E06-09-0876. 17192410 10.1091/mbc.E06-09-0876 PMC1805107

[67] Lokuta MA, Nuzzi PA, Huttenlocher A (2003) Calpain regulates neutrophil chemotaxis. Proc Natl Acad Sci U S A 100: 4006–4011 10.1073/pnas.0636533100. 12649322 10.1073/pnas.0636533100 PMC153038

[68] Stewart MP, McDowall A, Hogg N (1998) LFA-1-mediated adhesion is regulated by cytoskeletal restraint and by a Ca2+-dependent protease, calpain. J Cell Biol 140: 699–707. 9456328 10.1083/jcb.140.3.699 PMC2140165

[69] Kulkarni S, Saido TC, Suzuki K, Fox JE (1999) Calpain mediates integrin-induced signaling at a point upstream of rho family members. J Biol Chem 274: 21265–21275. 10409684 10.1074/jbc.274.30.21265

[70] Dedieu S, Mazeres G, Poussard S, Brustis JJ, Cottin P (2003) Myoblast migration is prevented by a calpain-dependent accumulation of MARCKS. Biol Cell 95: 615–623. 14720464 10.1016/j.biolcel.2003.09.005

[71] Dedieu S, Poussard S, Mazeres G, Grise F, Dargelos E, et al (2004) Myoblast migration is regulated by calpain through its involvement in cell attachment and cytoskeletal organization. Exp Cell Res 292: 187–200. 14720518 10.1016/j.yexcr.2003.08.014

[72] Louis M, Zanou N, Van Schoor M, Gailly P (2008) TRPC1 regulates skeletal myoblast migration and differentiation. J Cell Sci 121: 3951–3959 10.1242/jcs.037218. 19001499 10.1242/jcs.037218

[73] Lampe WR, Park J, Fang S, Crews AL, Adler KB (2012) Calpain and MARCKS protein regulation of airway mucin secretion. Pulm Pharmacol Ther. 10.1016/j.pupt.2012.06.003. 10.1016/j.pupt.2012.06.003 PMC3486950 22710197

[74] Bornfeldt KE, Raines EW, Graves LM, Skinner MP, Krebs EG, et al (1995) Platelet-derived growth factor. distinct signal transduction pathways associated with migration versus proliferation. Ann N Y Acad Sci 766: 416–430. 7486687 10.1111/j.1749-6632.1995.tb26691.x

[75] Uberall F, Giselbrecht S, Hellbert K, Fresser F, Bauer B, et al (1997) Conventional PKC-alpha, novel PKC-epsilon and PKC-theta, but not atypical PKC-lambda are MARCKS kinases in intact NIH 3T3 fibroblasts. J Biol Chem 272: 4072–4078. 9020116 10.1074/jbc.272.7.4072

[76] Hellberg C, Schmees C, Karlsson S, Ahgren A, Heldin CH (2009) Activation of protein kinase C alpha is necessary for sorting the PDGF beta-receptor to Rab4a-dependent recycling. Mol Biol Cell 20: 2856–2863 10.1091/mbc.E08-12-1228. 19369415 10.1091/mbc.E08-12-1228 PMC2695793

[77] Brown MV, Burnett PE, Denning MF, Reynolds AB (2009) PDGF receptor activation induces p120-catenin phosphorylation at serine 879 via a PKCalpha-dependent pathway. Exp Cell Res 315: 39–49 10.1016/j.yexcr.2008.09.025. 18950621 10.1016/j.yexcr.2008.09.025 PMC2925109

[78] Shiraishi M, Tanabe A, Saito N, Sasaki Y (2006) Unphosphorylated MARCKS is involved in neurite initiation induced by insulin-like growth factor-I in SH-SY5Y cells. J Cell Physiol 209: 1029–1038 10.1002/jcp.20814. 16941482 10.1002/jcp.20814

[79] Chappell DS, Patel NA, Jiang K, Li P, Watson JE, et al (2009) Functional involvement of protein kinase C-betaII and its substrate, myristoylated alanine-rich C-kinase substrate (MARCKS), in insulin-stimulated glucose transport in L6 rat skeletal muscle cells. Diabetologia 52: 901–911 10.1007/s00125-009-1298-7. 19252893 10.1007/s00125-009-1298-7 PMC2677811

[80] Beurden HE, Snoek PA, Hoff JW, Torensma R, Maltha JC, et al (2006) In vitro migration and adhesion of fibroblasts from different phases of palatal wound healing. Wound Repair Regen 14: 66–71 10.1111/j.1743-6109.2005.00090.x. 16476074 10.1111/j.1743-6109.2005.00090.x

[81] Kramer C, Nahmias Z, Norman DD, Mulvihill TA, Coons LB, et al (2008) Dermacentor variabilis: Regulation of fibroblast migration by tick salivary gland extract and saliva. Exp Parasitol 119: 391–397 10.1016/j.exppara.2008.04.005. 18492598 10.1016/j.exppara.2008.04.005

